# Characterization and transcriptome analysis of a dominant genic male sterile cotton mutant

**DOI:** 10.1186/s12870-020-02522-0

**Published:** 2020-07-03

**Authors:** Xin-Qi Cheng, Xin-Yu Zhang, Fei Xue, Shou-Hong Zhu, Yan-Jun Li, Qian-Hao Zhu, Feng Liu, Jie Sun

**Affiliations:** 1 Key Laboratory of Oasis Eco-agriculture, College of Agriculture, Shihezi University, Xinjiang, 832000 Shihezi China; 2 CSIRO Agriculture and Food, GPO Box 1700, Canberra, 2601 Australia

**Keywords:** *Gossypium hirsutum*, DEGs, Transcriptomic analysis, Pollen development, WGCNA

## Abstract

**Background:**

Male sterility is an efficient trait for hybrid seed production and germplasm innovation. Until now, most studies on male sterility were on cytoplasmic and recessive genic sterility, with few on dominant genic male sterility, especially in cotton, due to lack of such mutant.

**Results:**

We discovered a natural male sterile (MS) Sea Island cotton (*G. barbadense*) mutant. Genetic analysis showed the mutation was caused by a dominant mutation in a single nuclear gene. Comparative cytological observation of anther sections from MS and wild-type (WT) uncovered cellular differences in anther at and after the tetrad stage of pollen mother cells (PMC). In the MS anthers, the outer wall of pollen grains was free of spinules, the tapetum was vacuolated and showed delayed degradation, consequently, no functional pollen grains. Comparison of transcriptomes from meiosis, tetrad, mononuclear and binuclear pollen, and pollen maturation stages identified 13,783 non-redundant differentially expressed genes (DEGs) between MS and WT. Based on the number of DEGs, analyses of enriched GO terms and KEGG pathways, it was evident that significant transcriptomic changes occurred at and after the tetrad stage, consistent with cytological observation, and that the major differences were on metabolism of starch, sucrose, ascorbate, aldarate, alanine, aspartate and glutamate, and biosynthesis of cutin, suberine and wax. WGCNA analysis identified five modules containing 920 genes highly related to anther development, especially the greenyellow module with 54 genes that was highly associated with PMC meiosis and tetrad formation. A NAC transcription factor (*Gh_D11G2469*) was identified as a hub gene for this module, which warrants further functional characterization.

**Conclusions:**

We demonstrated that the MS trait was controlled by a single dominant nuclear gene and caused by delayed tapetum degradation at the tetrad stage. Comparative transcriptome analysis and gene network construction identified DEGs, enriched GO terms and metabolic pathways, and hub genes potentially associated with anther development and the MS trait. These results contribute to our understanding of dominant genic male sterility (DGMS) and provided source for innovation of cotton germplasm.

## Background

Male sterility generally refers to a biological trait in which monoecious plants maintain fully normal female functions, while male gametes cannot be produced. Adverse growth conditions, diseases or gene mutations can cause male sterility. Male sterility is typically divided into cytoplasmic sterility (CMS) and genic male sterility (GMS) [1]. CMS, caused by dysfunctional mitochondrial genes, shows typically non-Mendelian inheritance [2]. GMS, controlled by nuclear genes, is generally recessive mutations which affect a huge number of biological functions of plants [3]. Male sterility is an effective pollination control system and an important tool for hybrid seed production. Understanding the development of pollen and anther is essential for sexual reproduction. Many studies have probed physiological and biochemical changes during pollen and anther development in different plant species, and investigated the mechanisms of gene regulation and metabolism related to pollen development [4–8]. Some studies have identified key genes involved in pollen and anther development [9], such as *MALE STERILITY 2* (*MS2*), *CYP703A2*, and *CYP704B1* [10–12] that are involved in anther cell differentiation and division, pollen cell wall development, and anther dehiscence [13–18].

The formation of anthers undergoes a very complicated process. At the microsporocyte stage, the anther wall consists of four layers: epidermis, endothecium, middle layer and tapetum [19]. Proper programmed cell death (PCD) leading to controlled degradation of layers of anther wall is very important for the development of functional pollen grains. Tapetum is the most inner anther wall directly connecting with pollen mother cells (PMC), and its main function is to provide adequate nutrients for pollen development [20], thus, the development and timely degradation of the tapetum layer is critical for the development of pollen wall. Several genes have been shown to be involved in PCD and tapetum degradation. *MS1* encodes PHD (plant hemoedomain) transcription factor in *Arabidopsis.* The *ms1* mutant showed vacuolization of tapetum cells and delayed PCD, leading to male sterility [21, 22]. Many transcription factors, such as *DYT1, TDF1* and *AMS,* have been found to be involved in the regulation of tapetum degradation and development of anther wall. The *dyt1* mutant showed abnormal cell division and vacuolization of tapetum cells, resulting in formation of abnormal tetrads [23]. *TDF1* is a R2R3 *MYB* transcription factor and functions downstream of *DYT1*. The *tdf1* mutant showed vacuolized tapetum cells, leading to abnormal secretion and degradation of callose enzyme [24]. *AMS* is a basic helix-loop-helix (bHLH) transcription factor. The *ams* mutant showed vacuolization and delayed degradation of tapetum cells as well as defects in microspore outer wall, indicating that *AMS* is essential for tapetum cell development and microspore formation [25].

Cotton is the most important fiber crop and has distinct heterosis. However, use of hybrid vigor in cotton production is limited owning to lack of suitable male sterile materials for production of F1 seeds and high cost involved in production of F1 seeds by manual emasculation. Both CMS and GMS are available in cotton. In addition to genetic analysis of the MS mutants, physiological, biochemical, cytological and transcriptomic studies have been applied to investigate the MS mechanism. For instance, genes involved in hormone biogenesis and signaling, carbon and energy metabolism, and pollen wall development were found to be differentially expressed in different developmental stages of anthers from the “Dong A” MS mutant and its corresponding WT [26]. In 1355A, a GMS mutant caused by a single recessive mutation, the pollen wall of the mononuclear stage anther was thickened and had no spinules [27].

Despite the progress on both CMS and GMS studies in cotton, novel MS germplasm is desperately needed, particularly those stable GMS ones. In this study, we reported a new natural GMS mutant, which was found in the field of Sea Island cotton (*G. barbadense*). The sterile trait of the GMS mutant is controlled by a single dominant nuclear gene and has been introgressed into both elite *G. barbadense* and upland cotton (*G. hirsutum*) backgrounds. We further compared anther development and transcripome of Shida 98–6 (the recurrent upland cotton variety) and its nearly isogenic MS line Shida 98-6A to know the cellular phenotype of the MS mutant and the gene networks related to the MS trait.

## Results

### Genetic analysis of the male sterile trait

After discovery of the male sterile mutant, the male sterile trait has been transferred to Shida 98–6 (*G. hirsutum*) or Xinhai 53 (*G. barbadense*) background by five times of backcross using the sterile segregants as the female parent and Shida 98–6 or Xinhai 53 as the pollen donor. In each BC1F1 generation, we observed roughly equal number of sterile and fertile segregants. All progeny of the fertile segergants were fertile. These results suggest that the male sterile trait was caused by a single dominant nuclear gene that can only be maintained in heterozygotes. To further confirm this hypothesis, we generated several segregating populations by crossing sterile (designated Shida 98-6A or Xinhai 53A) or fertile BC5F1 segregant to the recurrent parent (Shida 98–6 or Xinhai 53) or another *G. hirsutum* variety Xinluzao33 (Table 1). The segregation of male fertility was analyzed based on anther/pollen analysis at the flowering stage and final boll setting. The BC6F1 population derived from backcrossing of Shida 98-6A and Shida 98–6 had 1015 sterile plants and 1004 fertile plants, consistent with 1:1 segregation ratio (χ^2^ = 0.06 < χ^2^_0.05_ = 3.84, *P* = 0.86 > 0.05). Similarly, the BC6F1 population derived from Xinhai 53A x Xinhai 53 and the two F1 populations derived from Shida 98-6A x Xinluzao33 and Xinhai 53A x Xinluzao33 all showed a 1:1 segregation ratio for sterile and fertile plants. In addition, the progeny of cross between the fertile BC5F1 Shida 98–6 plants and Shida 98–6 were all fully fertile (Table 1), suggesting that the fertile BC5F1 segregants are homozygotes. Together, these results indicate that the male sterility, which has been introgressed into Shida 98–6 or Xinhai 53, was caused by a dominant mutation in a single nuclear gene.

**Table 1:** Segregation of fertile and sterile plants in different backcross populations. ^#^ Sterile offsprings of backcrossing between Shida 98-6A and recurrent parent Shida 98–6 (BC5F1). ^&^ Sterile progeny of backcrossing between Xinhai53A and recurrent parent Xinhai53 (BC5F1). * Fertile offsprings from Shida 98-6A x recurrent parent Shida 98–6 (BC5F1)

Female	Male (Recurrent parent)	Total	Male sterility	Male fertility	Expected segregation	χ^2^ -value	p-value
Shida 98-6A^#^	Shida 98–6	2019	1015	1004	1:1	0.06	0.86
Xinhai53A^&^	Xinhai53	200	97	103	1:1	0.18	0.67
Shida 98-6A^#^	Xinluzao33	161	76	85	1:1	0.50	0.48
Xinhai53A^&^	Xinluzao33	192	95	97	1:1	0.02	0.89
Shida 98–6*	Shida 98–6	200	–	200	–	–	–

### Phenotypic characteristics of the male sterile mutant

**Fig. 1:**
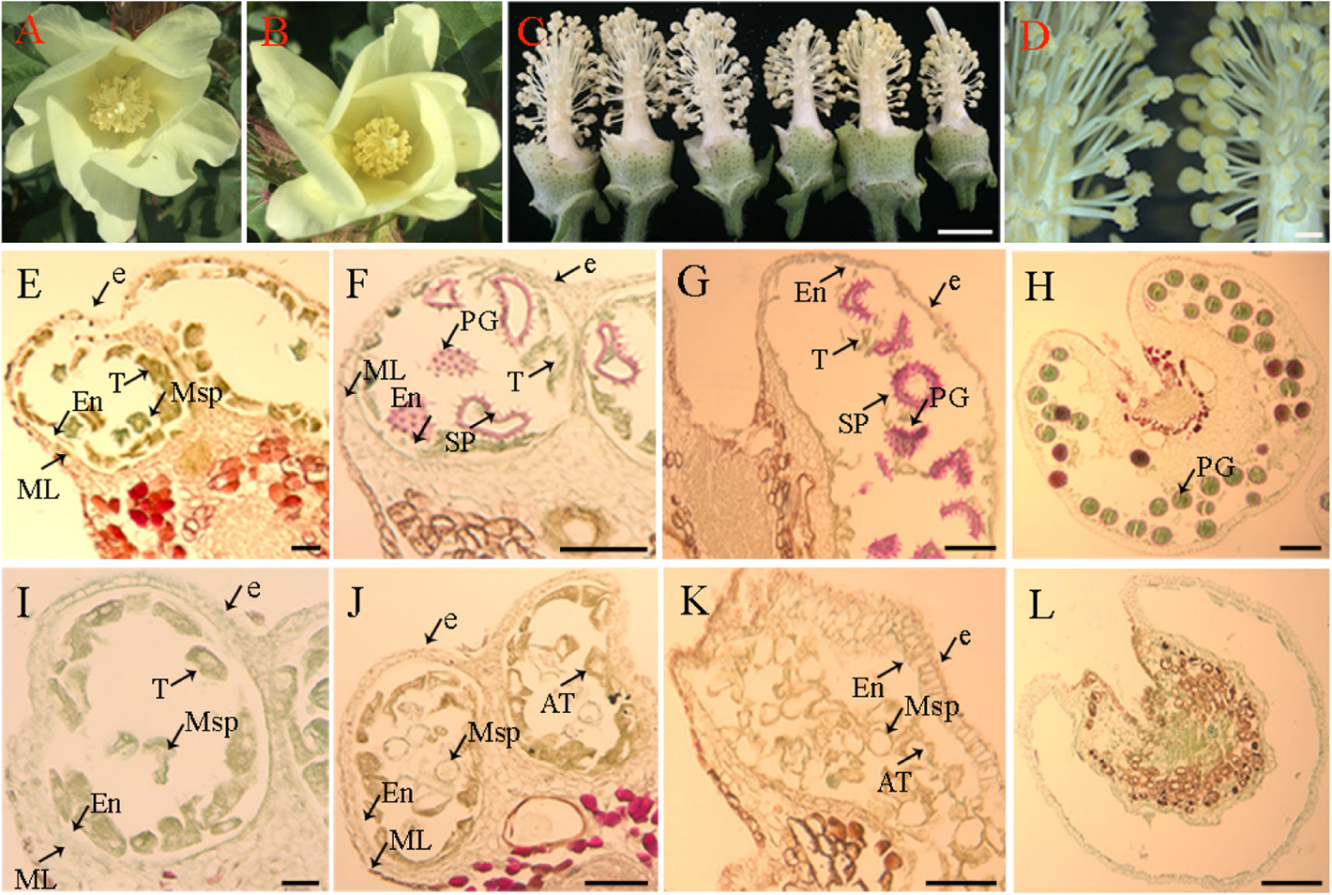
Phenotypic characteristics of flowers and microscopic observations of anthers **a.** 0 DPA wild-type flower. **b.** 0 DPA Shida 98-6A (MS) flower. **c.** 0 DPA flowers without petals, the left three and right three are from WT and MS plants, respectively. **d.** The anthers of fertile (left) and MS (right) segregants under the light microscope. **e-l.** Comparison of anther development in the WT and MS. **e.** Section of fertile anthers at meiosis stage. **f.** Section of fertile anthers at tetrad stage. **g.** Section of fertile anthers at mononuclear and binuclear pollen stage. **h.** Section of fertile anthers at pollen maturation stage. **i.** Section of male sterile anthers at meiosis stage. **j.** Section of male sterile anthers at tetrad stage. **k.** Section of male sterile anthers at mononuclear and binuclear pollen stage. **l.** Section of fertile male sterile anthers at pollen maturation stage. e, epidermis; ML, middle layer; En, endothecium; T, tapetum; Msp, microspore; AT, abnormal tapetum; SP, spinules protruding; PG, pollen grain. Scale bars in **c** and **d** represent 1 cm and 1000 μm, respectively; scale bars in **e** and **i** represent 20 μm; scale bars in **f, g, j** and **k** represent 50 μm; scale bars in **h** and **l** represent 100 μm

No morphological difference was observed between MS (Shida 98-6A) and WT (Shida 98–6) during vegetative growth and in the mature plants. During the period of reproductive growth, the MS and WT plants did not show noticeable difference in flowering time, petal color, flower bud size and other floral organs, but showed an obvious difference in anther appearance and development. The WT anthers were light yellow and normally dehisced to produce pollen grains at the mature stage (Fig. 1a). However, the MS anthers were dark yellow, and did not dehisce at the mature stage (Fig. 1b), and consequently no pollen grains were produced in MS (Fig. 1c, d). In order to identify the developmental differences in anthers between WT and MS, we dissected anthers and compared cytologically anther development between WT and MS at different developmental stages.

We determined the anther developmental stages based on optical microscope observation of paraffin sections according to the previous method [28]. The MS and WT anthers had similar cytological characteristics and no obvious differences in cellular structures at the meiosis stage of PMC (Fig. 1e, i). A clear defect in the MS anthers was first observed at the tetrad stage. At this stage in the WT anthers, microspore was released, the pollen grains were not full but crescent-shaped, and spinules protruding from the exine were formed, which were dyed pink by Safranine O (Fig. 1f), whereas in the MS anthers, microspore also released normally, however, development of microspore exine showed obvious abnormalities, including being unable to form spinules protruding and closely vacuolated tapetum cells (Fig. 1j). At the mononuclear and binuclear pollen stage, pollen grains of WT began to accumulate starch, which could be dyed green by Fast Green FCF, the outer walls of the pollen grains had normal spinules protruding, which were dyed pink by Safranine O, and the tapetum was absorbed and utilized as nutrients (Fig. 1g). In the MS mutant, the tapetum disintegrated and could not be completely absorbed by pollen grains as nutrients and showed a delayed degradation, and the microspores had no material accumulation as indicated by no staining by Fast Green FCF. Furthermore, pollen grains had no spinules protruding, could not be dyed pink by Safranine O, and finally aborted (Fig. 1k). At the mature stage, the WT pollen grains could be colored by Safranine O and Fast Green FCF (Fig. 1h), whereas no pollen grains but only the defective pollen wall cavities were observed in the MS anthers (Fig. 1l).

### Transcriptome analysis

To explore the molecular mechanism underlying anther abortion observed in the MS mutant, we compared transcriptomes of WT and MS anthers of the four developmental stages described in the last section, i.e. meiosis, tetrad, mononuclear and binuclear pollen, and pollen maturation stages, considering that the differences of anther cellular phenotypes started to be observed at the tetrad stage. In total, 24 libraries (2 genotypes × 4 developmental stages × 3 biological replicates) were sequenced and a total of 1,161,509,206 raw reads were generated. The sequence of nucleotide mass fraction Q30 greater than 30 was 92.94% in all samples, and the GC content was 43.22%. Raw reads were filtered to remove low quality ones and a total of 1,145,504,498 clean reads were finally used in alignment. Approximately 96.04% of the clean reads could be aligned to the TM-1 reference genome (see methods for details) with ~ 89.66% of them being uniquely aligned (Additional file 3: Table S1).

**Fig. 2:**
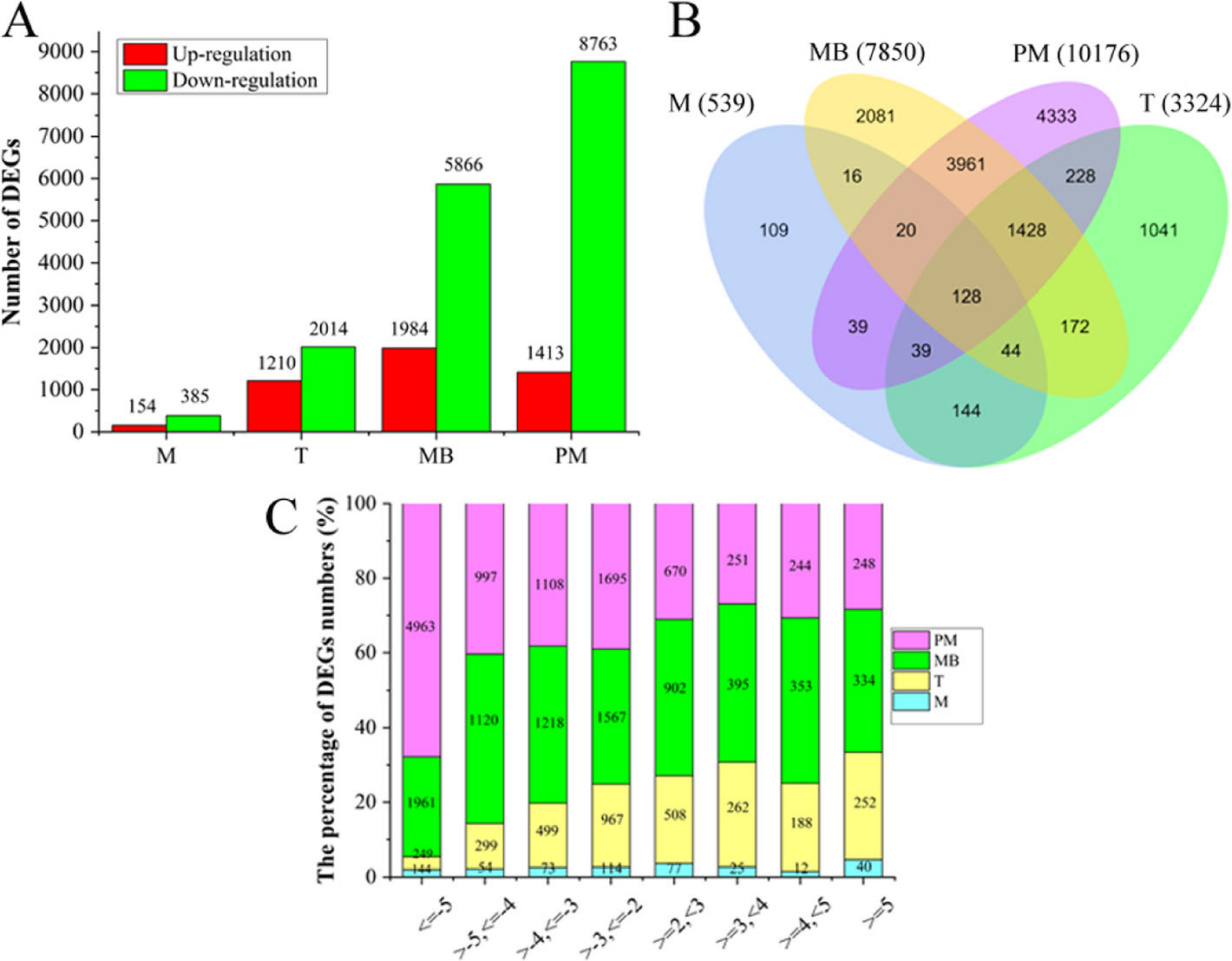
The number and distribution of differentially expressed genes between MS and WT. **a** The number of DEGs that were up-regulated or down-regulated in the four anther development stages. **b** Venn diagram showing DEGs overlapping in different anther development stages or unique to each developmental stage. **c** The distribution of DEGs (MS vs WT) with different fold changes (Log_2_ transformed). M, meiosis; T, tetrad; MB, mononuclear and binuclear pollen; PM, pollen maturation

In the four anther developmental stages, a total of 21,789 genes were found to be differentially expressed between MS and WT. Of those differentially expressed genes (DEGs), 17,028 (78.15%) were down-regulated and 4761 (21.85%) were up-regulated (|log_2_ (fold change) | ≥2 and *p*_adj_ < 0.05; Fig. 2a). Compared with WT, MS had a much higher number of down-regulated genes than that of up-regulated genes at all four developmental stages, especially at the two late stages (Fig. 2a). Of the 21,789 DEGs, 13,783 were unique ones with 109, 1041, 2081 and 4333 unique to meiosis, tetrad, mononuclear and binuclear pollen, and mature pollen stage, respectively, and 128 genes showed differential expression at all the four developmental stages (Fig. 2b). Of the up-regulated genes (MS vs WT), 2157 (45.31%) had a 2–3 folds change in expression while 874 (18.35%) had an over 5 folds change in gene expression. Among the down-regulated genes, 4343 (25.51%) had a 2–3 folds expression difference while 7317 (42.97%) had a > 5 folds expression difference (Fig. 2c).

### Confirmation of DEGs by qRT-PCR analysis

**Fig. 3:**
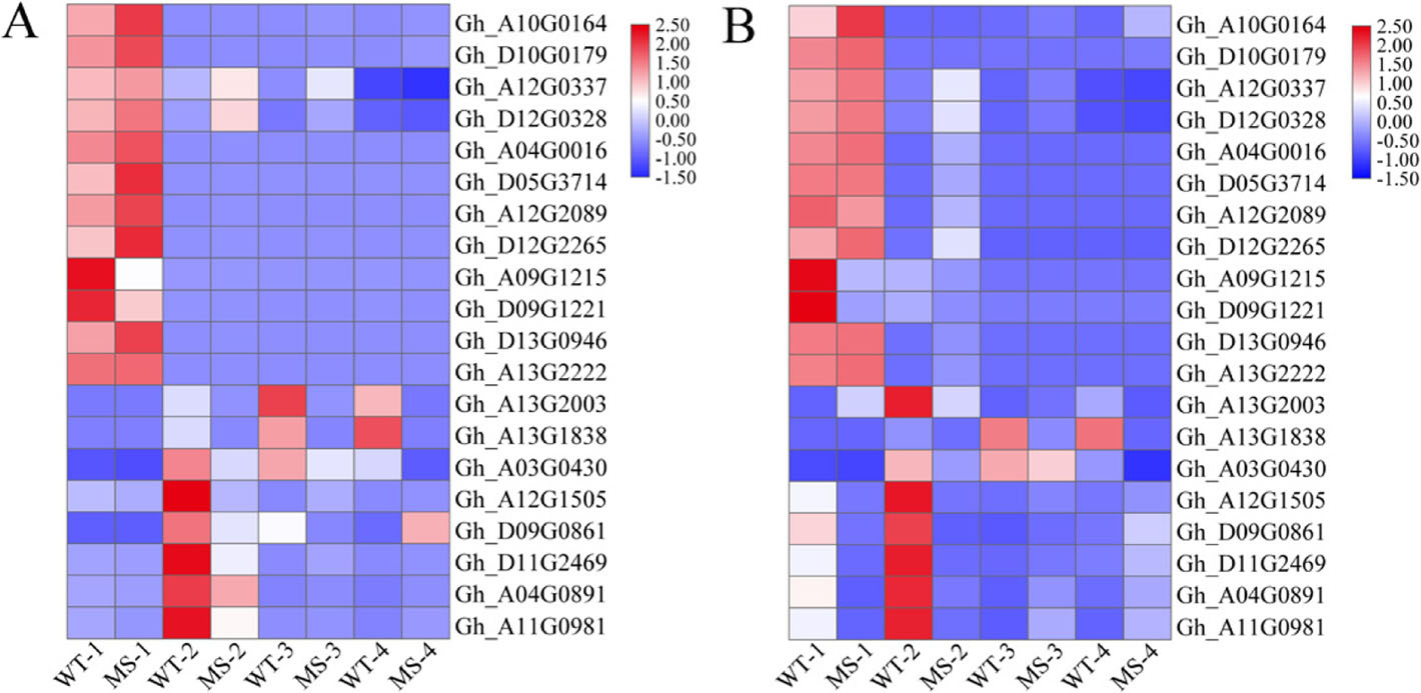
Heatmap showing the relative expression level of the 20 selected genes at the four anther developmental stages determined by qRT-PCR (a) and RNA-seq analysis (b). The 20 selected genes are 12 tapetal development related genes, *DYT1* (*Gh_A10G0164* and *Gh_D10G0179*), *AMS* (*Gh_A12G0337* and *Gh_D12G0328*), *MYB103* (*Gh_A04G0016* and *Gh_D05G3714*), *MS1* (*Gh_A12G2089* and *Gh_D12G2265*), *MS2* (*Gh_A09G1215* and *Gh_D09G1221*), and *TDF1* (*Gh_D13G0946* and *Gh_A13G2222*), and genes encoding ascorbate peroxidase (*Gh_A13G2003* and *Gh_A13G1838*), Ribosomal protein (*Gh_A03G0430*), NAC domain containing protein 47 (*Gh_D11G2469*), MLP-like protein 423 (*Gh_A04G0891*), myb domain protein 2 (*Gh_A11G0981*), NAC-like, activated by AP3/PI (*Gh_A12G1505*), and glutamine-dependent asparagine synthase 1 (*Gh_D09G0861*). MS: Shida 98-6A, WT: Shida 98–6. MS-1 and WT-1 represent meiosis stage, MS-2 and WT-2 represent tetrad stage, MS-3 and WT-3 represent mononuclear and binuclear pollen stage, and MS-4 and WT-4 represent pollen maturation stage

To validate the RNA-seq results and DEGs identified, we selected 20 genes for validation using quantitative real-time polymerase chain reaction (qRT-PCR), including 12 tapetum and anther development related genes, two genes (*Gh_A13G2003* and *Gh_A13G1838*) encoding ascorbate peroxidase, genes encoding NAC domain containing protein 47 (*Gh_D11G2469*), MLP-like protein 423 (*Gh_A04G0891*), myb domain protein 2 (*Gh_A11G0981*), NAC-like, activated by AP3/PI (*Gh_A12G1505*), glutamine-dependent asparagine synthase 1 (*Gh_D09G0861*) and ribosomal protein (*Gh_A03G0430*) (Fig. 3). Except *Gh_A13G2003*, all other genes showed the same trend of expression profiles between qRT-PCR (Fig. 3a) and RNA-Seq (Fig. 3b) in the four anther developmental stages, confirming the reliability of the RNA-seq results.

### Gene ontology analysis of DEGs

**Fig. 4:**
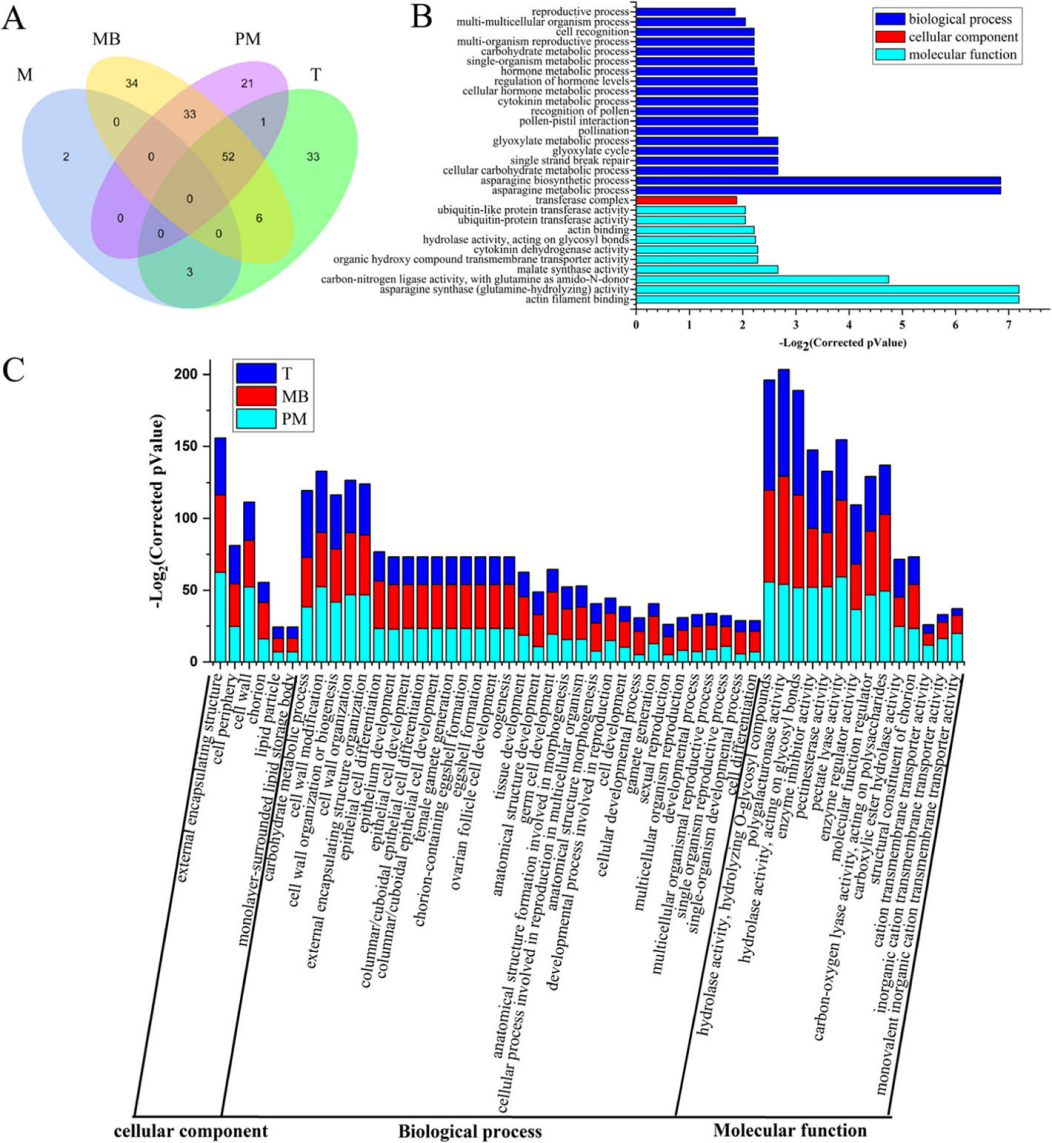
Gene ontology classification of DEGs between MS and WT. **a.** Venn diagram showing the number of enriched GO terms at the four anther developmental stages. **b.** Analysis of GO enrichment at meiosis stage. **c.** Analysis of GO enrichment at tetrad, mononuclear and binuclear pollen, and pollen maturation stages. M, meiosis; T, tetrad; MB, mononuclear and binuclear pollen; PM, pollen maturation

Gene Ontology (GO) analysis was performed using the DEGs identified between MS and WT in the four different anther developmental stages. Using the criterion of corrected *P*_Value_ ≤ 0.05, we found that the 13,783 DEGs were enriched for 118 GO terms (Additional file 4: Table S2), and identified 5, 95, 125 and 107 GO terms enriched at the meiosis, tetrad, mononuclear and binuclear pollen, and pollen maturation stage, respectively, with 2, 33, 34 and 21 GO terms unique to each of the corresponding stage (Fig. 4a). The number of enriched GO terms were significantly fewer at the meiosis stage (the top 30 GO terms instead of the 5 enriched ones were shown in Fig. 4b) than at the other three stages (Fig. 4c). Three GO terms were enriched at both the meiosis stage and the tetrad stage, 6 GO terms were enriched at both the tetrad stage and the mononuclear and binuclear pollen stage, 33 GO terms were enriched at both the mononuclear and binuclear pollen stage and the pollen maturation stage, and only 1 GO term was enriched at both the tetrad stage and the pollen maturation stage. A total of 52 GO terms were commonly enriched from the tetrad stage to the pollen maturation stage, and no commonly enriched GO term was found in all the four stages (Fig. 4a). The enriched GO terms were quite different between the meiosis stage and the other three stages (Additional file 5: Table S3). For example, the two biological process terms enriched at the meiosis stage were related to asparagine metabolism, whereas those enriched in the other three stages were mainly related to cell differentiation and cell wall organization or biogenesis (Fig. 4b, c). These results together with the number of DEGs identified at the four developmental stages suggest that the significant difference in anther development between MS and WT most likely starts from the tetrad stage, consistent with the cytological observation.

### KEGG pathways of DEGs

**Fig. 5:**
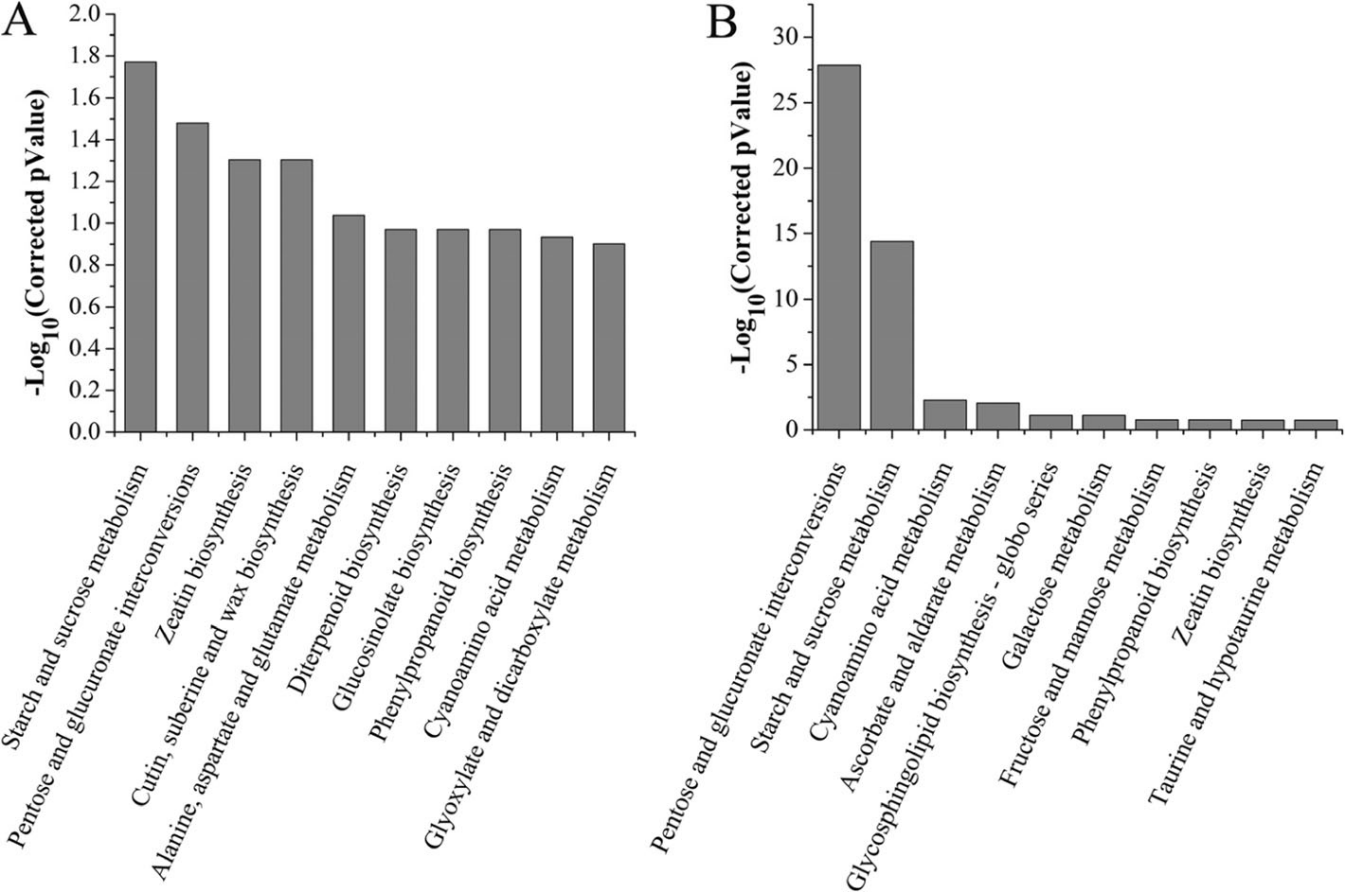
KEGG analysis of DEGs associated with anther developmental at meiosis and tetrad stages. **a** KEGG categories of DEGs associated at meiosis stage. **b** KEGGs categories of DEGs at tetrad stage. The horizontal axes show the top ten KEGG terms, the vertical axes show the logarithm (−log_10_) of the corrected P value

In order to further analyze the DEGs, we performed pathway analysis. Analysis of all the 13,783 non-redundant DEGs found 109 Kyoto Encyclopedia of Genes and Genomes (KEGG) pathways (Additional file 6: Table S4). Many significantly changed pathways were related to metabolism of starch and sucrose, plant-pathogen interaction, galactose metabolism, biosynthesis of endocytosis, sesquiterpenoid and triterpenoid, inositol phosphate metabolism, phenylpropanoid biosynthesis, and phagosome (Additional file 6: Table S4), many of them are associated with tapetum and pollen wall development. The enriched KEGG pathways at the meiosis stage included those involved in metabolism of starch, sucrose, alanine, aspartate and glutamate, interconversions of pentose and glucuronate, and biosynthesis of zeatin, cutin, suberine and wax (Fig. 5a). The DEGs involved in zeatin biosynthesis included genes encoding cytokinin oxidase/dehydrogenase (*Gh_D04G0688*) and don-glucosyltransferase (*Gh_D08G1604*). Among the DEGs involved in the metabolism of cutin, suberine and wax were genes encoding Jojoba acyl CoA reductase-related male sterility protein (*Gh_A09G1215*, *Gh_D09G1221*), cytochrome P450 (*Gh_D12G2271*) and caleosin-related family protein (*Gh_D10G1585*) (Additional file 6: Table S4, Additional file 1: Fig. S1B). At the tetrad stage, pathways related to metabolism of starch, sucrose, cyanoamino acid, ascorbate and aldarate, and interconversions of pentose and glucuronate were enriched (Fig. 5b), including genes encoding serine transhydroxy methyltransferase (*Gh_A11G1879*), BS-glucosidase (*Gh_D07G2431*, *Gh_D01G2109*) and ascorbate peroxidase (*Gh_A13G2003*) (Additional file 6: Table S4, Additional file 1: Fig. S1C). The pathways enriched at the mononuclear and binuclear pollen stage were related to metabolism of ether lipid and phenylalanine, including genes that encode phospholipid/glycerol acyltransferase (*Gh_A01G0955*) and peroxidase (*Gh_A08G2028* and *Gh_D11G0463*) (Additional file 6: Table S4, Additional file 1: Fig. S1D). The terpene synthase pathway with 21 DEGs, which is related to sesquiterpene and triterpene biosynthesis, was identified at the pollen mature stage (Additional file 6: Table S4, Additional file 1: Fig. S1E). These results suggest that anther development in cotton is controlled by a complex gene network regulating multiple metabolic pathways.

### Gene network analysis with WGCNA

**Fig. 6:**
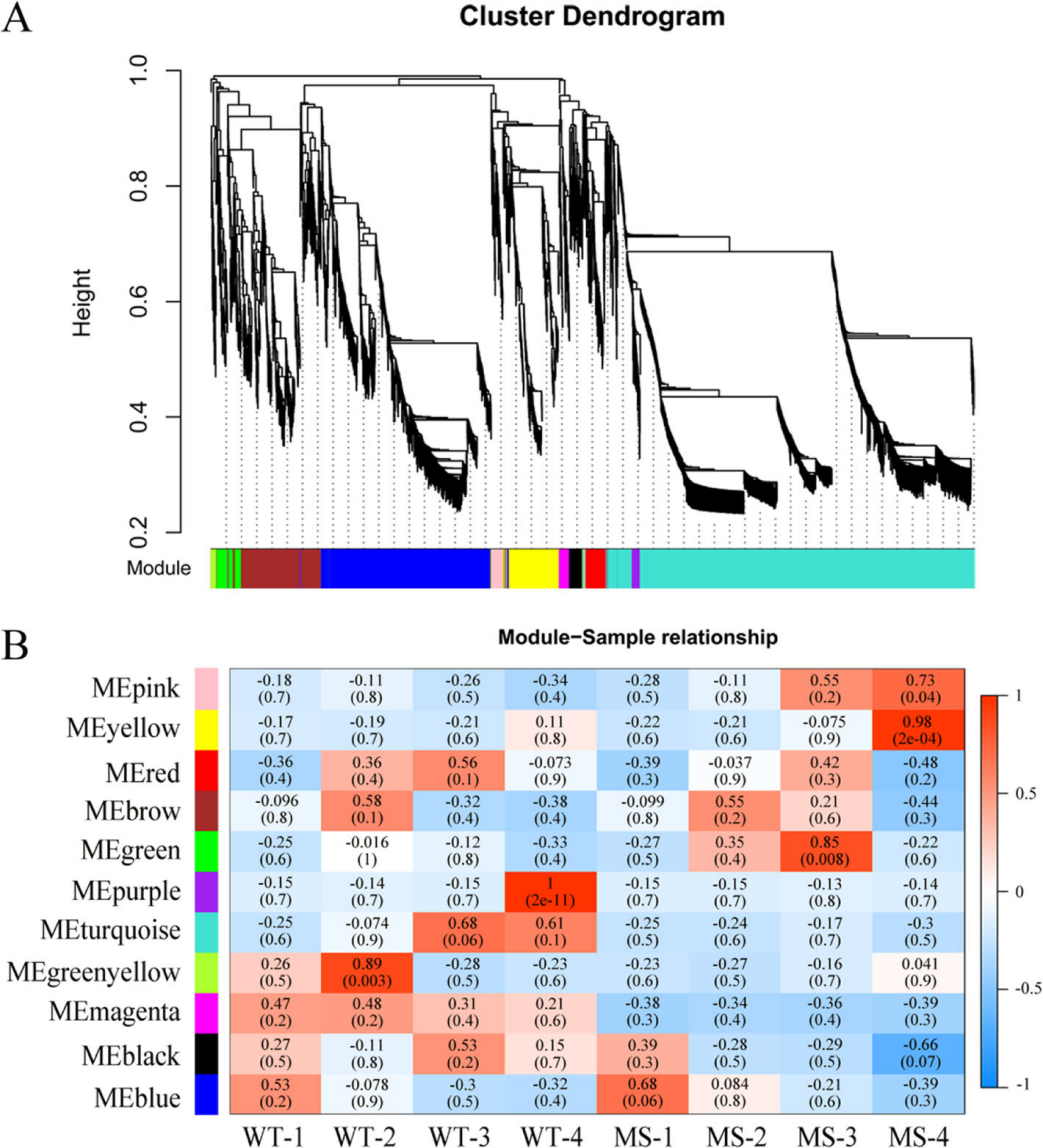
WGCNA of DEGs between MS and WT at each anther developmental stage. **a** Hierarchical cluster tree showing co-expression modules identified by WGCNA. The major tree includes 11 modules according to calculation of eigengenes; each module is highlighted in a designated colour. **b** Module-sample relationships. Each row corresponds to a different module showing on the left side, each column represents a sample, and the correlation coefficient and e-value of each sample-module relationship are displayed. Red represents high expression, and blue represents low expression. MS: Shida 98-6A; WT: Shida 98–6. MS-1 and WT-1 represent meiosis stage, MS-2 and WT-2 represent tetrad stage, MS-3 and WT-3 represent mononuclear and binuclear pollen stage, and MS-4 and WT-4 represent pollen maturation stage

Weighted Correlation Network Analysis (WGCNA) is a systematic biological approach for describing patterns of genes association with different samples. To identify the specific genes that are highly correlated with anther development, the co-expression networks were generated by WGCNA using the 13,783 non-redundant DEGs and all biological samples. A total of 11 gene modules associated with the specific expression profiles of different samples were identified (Fig. 6a). Of the 11 modules, 5 (greenyellow, green, purple, yellow and pink) were significantly associated with tetrad, mononuclear and binuclear pollen, or pollen maturation stage in WT or MS, and no gene module was significantly associated with the meiosis stage although the blue module was marginally significant in MS (Fig. 6b). The greenyellow module with 54 genes was highly associated with the tetrad stage of WT (Shida 98–6). The green (191 genes) and turquoise (3262 genes) modules were significantly and marginally significantly associated with the mononuclear and binuclear pollen stage in MS and WT, respectively. There were 78 genes in the purple module that was significantly associated with the pollen maturation stage of WT. The yellow (492 genes) and pink (105 genes) modules were significantly associated with the pollen maturation stage in MS in addition to the marginally significant black module (Fig. 6b).

The 54 DEGs of the greenyellow module were enriched for 10 GO terms, including asparagine synthase (glutamine-hydrolyzing) activity, asparagine metabolic process, asparagine biosynthetic process, aspartate family amino acid biosynthetic process and aspartate family amino acid metabolic process (Additional file 2: Fig. S2A). At the meiosis and tetrad stages, the greenyellow module was highly associated with alanine, aspartate and glutamate metabolism (Corrected *P*_*Value*_ = 9.15 × 10^− 3^, 3 genes) and diterpenoid biosynthesis (Corrected *P*_*Value*_ = 1.38 × 10^− 2^, 2 genes) (Additional file 2: Fig. S2B). The KEGG pathway analysis was also performed on the genes in each of other modules (Additional file 7: Table S5), and their fragments per kilobase of transcript per million mapped reads (FPKM) values were shown in Additional file 8: Table S6.

### Construction of co-expression gene networks and identification of hub genes associated with male sterility

**Fig. 7:**
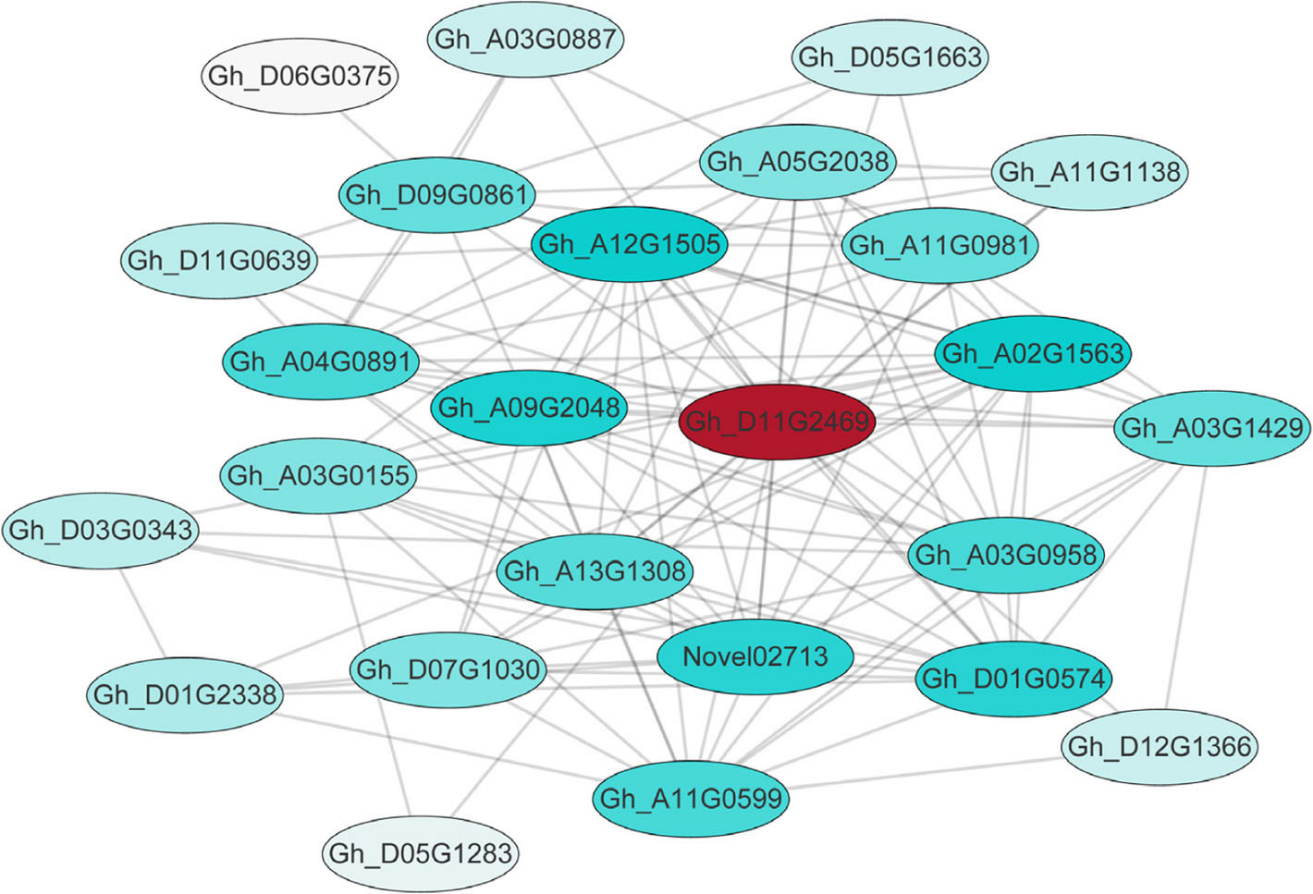
Co-expression network analysis of the greenyellow module. Red circle represented the hub gene and transcription factor, the light blue circle indicates the hub gene, and the color shade indicates the correlation

According to cytological observation, the difference in anther development between WT and MS first appeared at the tetrad stage, our focus was thus on the greenyellow module as it was significantly associated with the tetrad stage in WT and most of its genes were significantly down-regulated at the tetrad stage in MS (Fig. 6b). Among these down-regulated genes, our focus was further on those that were also down-regulated during meiosis, as they might be related to the degradation of the tapetum and the formation of pollen grains in the early stage. In the greenyellow module, we identified 25 hub genes based on the criteria of eigengene-based connectivity (*K*_*ME*_) value ≥0.9 and edge weight value ≥0.4. The major hub genes included genes encoding NAC domain containing protein 47 (*Gh_D11G2469*), MLP-like protein 423 (*Gh_A04G0891*), myb domain protein 2 (*Gh_A11G0981*) and glutamine-dependent asparagine synthase 1 (*Gh_D09G0861*) (Fig. 7, Table 2).

**Table 2:** Candidate hub genes in the greenyellow module

Gene ID	*Arabidopsis* ID	Function Description	*K*_*ME*_
*Gh_D11G2469*	*AT3G04070*	NAC domain containing protein 47	0.99678203
*Gh_A04G0891*	*AT1G24020*	MLP-like protein 423	0.991487505
*Gh_A11G0981*	*AT2G47190*	myb domain protein 2	0.988614125
*Gh_A09G2048*	*AT4G15610*	Uncharacterised protein family (UPF0497)	0.986062006
*Gh_A12G1505*	*AT1G69490*	NAC-like, activated by AP3/PI	0.985852055
*Gh_D09G0861*	*AT3G47340*	glutamine-dependent asparagine synthase 1	0.984832341
*Gh_D11G0639*	*AT5G13870*	xyloglucan endotransglucosylase/hydrolase 5	0.975723499
*Gh_A03G1429*	*AT5G54510*	Auxin-responsive GH3 family protein	0.974560042
*Gh_A02G1563*	*AT4G36740*	homeobox protein 40	0.96720103
*Gh_A11G0599*	*AT5G20090*	Uncharacterised protein family (UPF0041)	0.963665581
*Gh_D06G0375*	*AT1G13700*	6-phosphogluconolactonase 1	0.958814873
*Gh_A11G1138*	*AT1G03120*	responsive to abscisic acid 28	0.955938829
*Gh_D05G1663*	*AT1G05010*	ethylene-forming enzyme	0.954645698
*Gh_D12G1366*	*AT1G11360*	Adenine nucleotide alpha hydrolases-like superfamily protein	0.953952827
*Gh_A03G0887*	*AT1G69490*	NAC-like, activated by AP3/PI	0.952575437
*Gh_A03G0958*	*AT3G21720*	isocitrate lyase	0.95257129
*Gh_D01G0574*	*AT1G11530*	C-terminal cysteine residue is changed to a serine 1	0.949804194
*Gh_A13G1308*	*AT1G78440*	*Arabidopsis thaliana* gibberellin 2-oxidase 1	0.948565051
*Novel02713*	*–*	Uncharacterised protein	0.947702632
*Gh_D05G1283*	*AT3G13750*	beta galactosidase 1	0.945131864
*Gh_A03G0155*	*–*	Uncharacterised protein	0.941948443
*Gh_D07G1030*	*AT3G47340*	glutamine-dependent asparagine synthase 1	0.940100681
*Gh_A05G2038*	*AT1G21400*	Thiamin diphosphate-binding fold (THDP-binding) superfamily protein	0.93916608
*Gh_D01G2338*	*AT1G78850*	D-mannose binding lectin protein with Apple-like carbohydrate-binding domain	0.937479802
*Gh_D03G0343*	*AT5G65140*	Haloacid dehalogenase-like hydrolase (HAD) superfamily protein	0.936073889

### Tapetum and anther development related genes

Tapetum plays important roles in pollen development. Any abnormality in tapetum development will lead to pollen abortion. In *Arabidopsis*, a number of genes and transcription factors, including *DYT1, TDF1, AMS, MYB103* and *MS1,* regulating development of tapetum have been reported [29]. In our anther transcriptome data, the cotton orthologs of these genes were clustered in the blue module (Fig. 6b), which was marginally associated with the meiosis stage in MS (Fig. 3b). Individually, these tapetum development related genes were not differentially expressed at the meiosis stage between WT and MS, but were significantly up-regulated at the tetrad stage in MS (Fig. 3b), suggesting that these genes might contribute to the MS trait although they might not be the cause of the MS trait. Several cotton orthologs of other Arabidopsis and rice genes related to anther and tapetum development were also differentially expressed at the meiosis and/or tetrad stages. For instance, the *MS2* genes (*Gh_A09G1215*, *Gh_D09G1221*) encoding the Jojoba acyl CoA reductase-related male sterility protein (Fig. 3b) were down-regulated at the meiosis and tetrad stages in MS compared to WT, while *ACOS5* (*Gh_A03G1091*, *Gh_D02G1514*) encoding acyl-CoA synthetase 5, and *CYP703A1* (*Gh_A12G1506*, *Gh_D12G2768*) and *CYP703A2* (*Gh_A12G1014*, *Gh_D12G1132*) encoding cytochrome P450 were up-regulated during the tetrad stage in MS anthers (Fig. 3b).

## Discussion

Male sterility prevents autogamy and permits allogamy, and thus reduces the cost of hybrid seed production by eliminating the process of emasculation. The male sterile trait described in this study was controlled by a dominant mutation in a single nuclear gene (Table 1), which adversely affected anther and pollen development, resulting in complete pollen abortion (Fig. 1). The MS mutation was stable in different genetic backgrounds and under variable environmental conditions, it would be a highly valuable trait in cotton heterosis breeding. In breeding, recurrent selection is an effective strategy to improve the genetic structure of crop population and improve the frequency of favorable genes in the population through multiple hybridization and selection. It plays an important role in crop improvement as the favorable genes of different germplasm could be concentrated in one individual by recurrent selection [30–32]. We have introgressed the MS trait into different elite cotton genetic backgrounds, such as Shida 98–6 and Xinhai 53. The new MS germplasm provides a powerful tool for creating new germplasm resources because a large number of crosses could be easily performed by using the elite MS introgression germplasm as female parents.

The MS mutant did not produce any pollen grains, which could be caused by developmental defects between the meiosis stage and the tetrad stage as the initial anther developmental differences between MS and WT were observed at the tetrad stage (Fig. 1f, j). At the tetrad stage, the tapetum of MS anther became vacuolated. At the mononuclear and binuclear stage, compared to the WT anther, the MS anther had more tapetum residue or remaining tapetum cells, the pollen wall became thickened (Fig. 1k), and pollen grains had no spinules (Fig. 1j, k). Tapetum is the innermost anther wall directly connected with pollen mother cells, playing an important role in pollen development. With the formation of microspores, tapetum cells began to functionally differentiate to form cells with secretory function. The degradation of tapetum provides necessary enzymes and nutrients for microspore development [33, 34]. Tapetum degradation has thus to be very precisely regulated, and precocious or delayed degradation of tapetum could lead to microspore abortion [35]. Therefore, we speculate that the delayed tapetum degradation or failure in development of functional pollen grains might be the underlying cause of the MS trait we discovered.

It had been reported that mutations of transcription factor genes regulating tapetum development could lead to tapetum cavitation, tapetum irregular degradation and pollen wall defects in *Arabidopsis thaliana* [36]. A gene network or cascade, *DYT1* - *TDF1* - *AMS* - *MYB103* - *MS1*, has been proposed to be involved in regulation of the development and degradation of tapetum in *Arabidopsis* [21, 34]. *TDF1* is a R2R3 *MYB* transcription factor gene. In *A. thaliana*, tapetum cells of the *tdf1* mutant had serious morphological vacuoles and could not transit to secretory tapetum [37]. Its rice ortholog (*OsTDF1*) played a role similar to that of *A. thaliana* [38]. Mutation in *OsTDF1* resulted in changes in hydrolytic enzyme, hydrophobic protein, cellulose, hemicellulose and pectin polymer, which play a role in the development of pollen wall. In the MS anther, the expression level of the cotton orthologs of Arabidopsis *TDF1* and other genes of the network regulating tapetum development and degradation were up-regulated at the tetrad stage, suggesting on-going degradation of tapetum at this stage in the MS anther, consistent with the observed phenotype of delayed degradation of tapetum in the MS anther (Fig. 1j, k).

In rice and *A. thaliana*, Reactive oxygen species (ROS) regulates PCD in the tapetum, thereby affecting the development of male gametes [39, 40]. To maintain the dynamic balance of ROS, plants contain enzymes that produce ROS (such as NADPH oxidase and peroxidase) and enzymes that remove ROS (such as ascorbate peroxidase and glutathione peroxidase). Antioxidants, such as ascorbic acid and glutathione mainly act on scavenging hydroxyl radicals and singlet oxygen [41]. Ascorbic acid and glutathione related genes were enriched in DEGs at the tetrad stage based on KEGG analysis (Fig. 5b) and the expression level of genes was lower in MS than in WT (Additional file 1: Fig. S1C), which might lead to the failure of scavenging of ROS in cells of the MS mutant. Consequently the ROS homeostasis could not be maintained in cells of the MS mutant, leading to destroying the critical period of anther development and the occurrence of plant sterility. GO analysis showed that asparagine synthase (glutamine-hydrolyzing) activity, asparagine biosynthetic and metabolic process were enriched at the meiosis stage and the greenyellow module (Fig. 4b, Additional file 2: Fig. S2A), and KEGG analysis found enriched alanine, aspartate and glutamate metabolism in the greenyellow module (Additional file 2: Fig. S2).

Based on the WGCNA analysis (Table 2) and the correlation networks map (Fig. 7), a gene encoding NAC domain containing protein (*Gh_D11G2469*) was identified as the candidate hub gene highly associated with anther development at the meiosis and tetrad stages. *Gh_D11G2469* is homologous to *Arabidopsis* genes *AT1G61110* and *AT3G04070*. *AT1G61110* is specifically expressed in tapetum based on GUS fusion analysis. Mutation of the gene led to defects in tapetum and pollen development, and finally male sterility [42]. The functionality of *AT3G04070* has not been reported. Down-regulation of the rice homologue, *Os07g37920*, of *Gh_D11G2469* reduced the proportion of viable pollen grains and caused male sterility [43]. Considering these demonstrated roles of *Arabidopsis* and rice homologues in anther and pollen development, the role of *Gh_D11G2469* in cotton anther and pollen development warrants further characterization.

## Conclusions

We described a natural male sterile mutation found in Sea Island cotton, which is controlled by a single dominant nuclear gene. Comparison of sterile and fertile anthers demonstrated cytologically that male sterile was due to pollen abortion caused by developmental defects during the transition from meiosis to tetrad formation. Comparative transcriptome analysis supported the cytological observations. Gene network and pathway analyses identified gene modules and candidate genes regulating anther and pollen development. The MS trait has been introgressed into elite *G. hirsutum* and *G. barbadense* backgrounds, providing novel MS germplasm for developing cross combinations with hybrid vigor, production of hybrid seeds, and innovation of germplasm resource.

## Methods

### Plant materials

In the summer of 1992, we found a male sterile Sea Island cotton plant (*G. barbadense*). Flowers of the sterile plant were pollinated with pollens from fertile plants to preserve the sterile trait. The F1 population showed roughly equal number of fertile and sterile plants, and all F2 progeny derived from the fertile F1 plants were fertile, indicating that the male sterile trait is controlled by a single dominant genic mutation. The male sterility mutant used in this study was provided by the Cotton Research Institute of Shihezi University. First, the male sterility of sea island cotton was transferred to upland cotton through backcross generations, the sterile trait was then introgressed into Upland cotton Shida 98–6 (*G. hirsutum*) and Sea Island cotton Xinhai 53 (*G. barbadense*) background by backcross (six times) to breed nearly isogenic male sterile line Shida 98-6A and Xinhai 53A, respectively. Several populations, including two derived from cross Shida 98-6A or Xinhai 53A with another Upland cotton variety Xinluzao 33, were generated for genetic analyses (Table 1). The segregating populations were grown in the experimental field of the Cotton Institute of Shihezi University in Xinjiang, China. The plants (WT and MS) used in microscope observation and transcriptome analysis were grown in artificial climate chamber at 28–30 °C with a 16 L: 8 D photoperiod scheme and 60% relative humidity.

### Histological analyses

Different sizes of flower buds with a diameter < 9 mm were harvested from MS and WT plants to identify anthers at the following four developmental stages: meiosis, tetrad, mononuclear and binuclear pollen and pollen maturation stages using optical microscopy [28]. The anthers of the four developmental stages were separately collected, frozen in liquid nitrogen and stored at − 80 °C for subsequent experiments. For histological observation, the samples were fixed in FAA (5% formalin, 5% glacial acetic acid, 70% alcohol), dehydrated with increasing concentration of alcohol (70, 85, 95, 100%) for 1 h each, and then treated with increasing concentration of dimethylbenzene (1/2 alcohol + 1/2 dimethylbenzene, 1/4 alcohol + 3/4 dimethylbenzene and pure dimethylbenzene) for 1 h each, and finally soaked and embedded in wax. After embedding, the samples were sliced to sections with a thickness of 8 μm. A drop of sticky tablet sticks and spreads were dropped on a clean glass slide with the sliced sections, and baked and dried. The samples were dewaxed stained by 1% Safranine O (configured with 85% alcohol) and 1% Fast Green FCF method (configured with 95% alcohol), and then dehydrated, transparent, and mounted for observation using optical microscopy.

### RNA extraction, library construction, and RNA-Seq

The anthers collected at different stages were sent to Beijing Novogene bioinformatics technology Co., Ltd. for RNA-Seq. The purity and integrity of the RNA samples were determined by NanoPhotometer Spectrophotometer and Agilent 2100 Bioanalyzer. RNA integrity and possible contamination were further determined using 1% agarose gel electrophoresis. A total of 3 μg total RNA per sample was used in RNA-seq library preparation (each developmental stage with three biological replicates). Sequencing was done using an Illumina HiSeq 4000 sequencing platform with 150 base pair (bp) paired-end (PE) reads.

### Data processing

Raw data in FASTq format were processed using Perl scripts to determine the quality of the data, including the percentage of GC content, Q20 and Q30. Low-quality reads were filtered and eliminated to obtain clean reads for subsequent analysis. Indexes of the *G. hirsutum* reference genome were built by Bowtie v2.2.3 and the gene model annotation files were downloaded from the CottonGen database (http://www.cottongen.org). The generated clean reads were mapped to the *G. hirsutum* TM-1 genome [44] using TopHat v2.0.12. The Cufflinks software was used to calculate the expression levels of individual genes using FPKM [45]. The DESeq software was used to determine differential gene expression through negative binomial distribution and calculation of false discovery rate (FDR) by the Benjamini and Hochberg method, as well as the adjusted *p*_*value*_. The adjusted *p*_*value*_ < 0.05 was used as the threshold for identification of DEGs among samples.

### Function annotation of DEGs and KEGG pathways

The functions of DEGs were annotated based on homology with the annotated *Arabidopsis* genes using TAIR (*Arabidopsis* information resources). GOseq [46] was used to perform GO functional enrichment analysis of DEGs. KEGG enrichment was done by the KOBAS 2.0 software.

### Gene network construction and screening of hub genes

The WGCNA R package [47] was used to construct the co-expression networks associated with anther development. The hub genes were screened on the basis of the module *K*_*ME*_ values and high-weight values. The correlation networks were drawn using Cytoscape 3.6.1.

### Quantitative RT-PCR validation of DEGs

Quantitative RT-PCR (qRT-PCR) was performed to validate DEGs according to previously reported protocol [48]. Gene-specific primers were designed according to the cDNAs sequences with NCBI Primer-BLAST (https://www.ncbi.nlm.nih.gov/tools/primer-blast/) (Additional file 9: Table S7). Total RNA was extracted from the samples using RNA extraction kit (Takara). And then total RNA (1 μg) was denatured and reverse transcribed by using PrimeScript™ RT reagent Kit with gDNA Eraser (Takara) at 42 °C for 15 min, 58 °C for 5 s. qRT-PCR was performed on Light Cycler® 480II, using Power SYBR Green PCR Master Mix, and 10 μl PCR reactions contained 1 μl of cDNA, 5 μl of SYBR Green PCR Master Mix, 10 μM of each pair of target primers and 3 μl of H_2_O. PCR conditions were as follows: pre-incubation at 95 °C for 5 min, followed by 40 cycles of 94 °C for 15 s, 58 °C for 20 s, and 72 °C for 20 s, then melting curve with 95 °C for 5 s, 65 °C for 1 min, 97 °C for 1 min, finally cooling at 40 °C for 10 s. Three technical replicates from three independent biological experiments were performed for qRT-PCR analyses. The relative expression levels were analyzed according to the 2^−∆∆C^_T_ method and ubiquitin (*GhUBI*, XM_012634824) was used as a reference gene [49].

## Supplementary information

**Additional file 1: Figure S1.** Heatmap comparison DEGs associated with anther developmental stages. **a.** Venn diagram showed the different KEGGs in four stages. **b, c, d** and **e** showed the DEGs associated to the functional categories at meiosis stage, tetrad stage, mononuclear and binuclear pollen stage and pollen maturation stage, respectively. Red represents high expression, and blue represents low expression. Each row represents a DEG.

**Additional file 2: Figure S2.** GO analysis and KEGG pathways of the greenyellow module genes. **a.** Analysis of GO enrichment of the greenyellow module genes. * represented significant enrichment (Corrected *P*_*Value*_ ≤ 0.05). **b.** KEGG categories of DEGs at the greenyellow module. The horizontal axis was rich factor, The ertical axis was statistics of pathway enrichment. Circle size represented the number of genes. Red represents high KEGG enrichment, and blue represents low KEGG enrichment.

**Additional file 3: Table S1.** Transcriptome sequencing data quality and genome mapping.

**Additional file 4: Table S2.** Analysis of GO enrichment for 13,783 common DEGs.

**Additional file 5: Table S3.** Analysis of GO enrichment from tetrad stage to pollen maturation stage.

**Additional file 6: Table S4.** Analysis of KEGG enrichment of the DEGs.

**Additional file 7: Table 5.** Analysis of KEGG enrichment in green, purple, yellow and pink modules.

**Additional file 8: Table S6.** Key KEGG pathways genes enriched in five module.

**Additional file 9: Table S7.** The primers used for qRT-PCR.

## Data Availability

All Gene ID and annotation files could be obtained from CottonGen (https://www.cottongen.org). Raw data and other data generated or analyzed were included in this published article in this study (Additional file 1, Additional file 2, Additional file 3, Additional file 4, Additional file 5, Additional file 6, Additional file 7, Additional file 8, Additional file 9). The datasets used and/or analysed have been deposited in the National Center for Biotechnology Information (NCBI). The accession number is PRJNA640994 (https://www.ncbi.nlm.nih.gov/bioproject/PRJNA640994), which includes 24 accession items (SRR12065069 – SRR12065092).

## Abbreviations

CMS: Cytoplasmic sterility
GMS: Genic male sterility
DGMS: Dominant genic male sterility
PMC: Pollen mother cells
bHLH: Basic helix-loop-helix
PHD: Plant hemoedomain
PCD: Programmed cell death
DEGs: Differentially expressed genes
qRT-PCR: Quantitative real-time polymerase chain reaction
GO: Gene Ontology
KEGG: Kyoto Encyclopedia of Genes and Genomes
WGCNA: Weighted Correlation Network Analysis
*K*_*ME*_: Eigengene-based connectivity
FPKM: Fragments per kilobase of transcript per million mapped reads
ROS: Reactive oxygen species
PE: Paired-end
FDR: False discovery rate
WT: Fertile line (Shida 98–6)
MS: Male-sterile mutant line (Shida 98-6A)

## References

[1] Jenny C, Matti L, Joel S, Jens FS, Kristina G (2008). Mitochondrial regulation of flower development. Mitochondrion..

[2] Zhang HG, Cheng XJ, Zhang LJ, Liu QQ, Gu MH, Tang SZ (2019). Identifying the genes around Rf5 and Rf6 loci for the fertility restoration of WA-type cytoplasmic male sterile japonica rice (*Oryza sativa*) lines. Euphytica..

[3] Zhang YJ, Chen J, Liu JB, Xia MX, Wang W (2015). ShenFF. Transcriptome analysis of early anther development of cotton revealed male sterility genes for major metabolic pathways. J Plant Growth Regul.

[4] Liu ZH, Shi XY, Li S, Zhang LL, Song XY (2018). Oxidative stress and aberrant programmed cell death are associated with pollen abortion in isonuclear alloplasmic male-sterile wheat. Front Plant Sci.

[5] Engelke T, Hirsche J, Roitsch T (2010). Anther-specific carbohydrate supply and restoration of metabolically engineered male sterility. J Exp Bot.

[6] Edstam MM, Edqvist J (2014). Involvement of GPI-anchored lipid transfer proteins in the development of seed coats and pollen in *Arabidopsis thaliana*. Physiol Plantarum.

[7] Hsieh K, Huang AHC (2007). Tapetosomes in Brassica tapetum accumulate endoplasmic reticulum–derived flavonoids and alkanes for delivery to the pollen surface. Plant Cell.

[8] Chen GH, Ye XY, Zhang SY, Zhu SD, Yuan LY, Hou JF, Wang CG (2018). Comparative transcriptome analysis between fertile and CMS flower buds in Wucai (*Brassica campestris* L.). BMC Genomics.

[9] Wilson ZA, Zhang DB (2009). From *Arabidopsis* to rice: pathways in pollen development. J Exp Bot.

[10] Chen WW, Yu XH, Zhang KS, Shi JX, Oliveira SD, Schreiber L, Shanklin J, Zhang DB (2011). *Male sterile 2* encodes a plastid-localized fatty acyl carrier protein reductase required for pollen exine development in *Arabidopsis*. Plant Physiol.

[11] Shi J, Tan HX, Yu XH, Liu YY, Liang WQ, Ranathunge K, Franke RB, Schreiber L, Wang YJ, Kai GY, Shanklin J, Ma H, Zhang DB (2011). Defective pollen wall is required for anther and microspore development in rice and encodes a fatty acyl carrier protein reductase. Plant Cell.

[12] Xu J, Ding ZW, Barrena GZ, Shi JX, Liang WQ, Yuan Z, Werck-Reichhart D, Schreiber L, Wilson ZA, Zhang DB (2014). *ABORTED MICROSPORES* acts as a master regulator of pollen wall formation in *Arabidopsis*. Plant Cell.

[13] Wijeratne AJ, Zhang W, Sun YJ, Liu WL, Albert R, Zheng ZG, Oppenheimer DG, Zhao DZ, Ma H (2007). Differential gene expression in *Arabidopsis* wild-type and mutant anthers: insights into anther cell differentiation and regulatory networks. Plant J.

[14] Thorstensen T, Grini PE, Mercy IS, Alm V, Erdal S, Aasland R, Aalen RB (2008). The *Arabidopsis* SET-domain protein ASHR3 is involved in stamen development and interacts with the bHLH transcription factor *ABORTED MICROSPORES* (*AMS*). Plant Mol Biol.

[15] Lou Y, Xu XF, Zhu J, Gu JN, Blackmore S, Yang ZN (2014). The tapetal AHL family protein TEK determines nexine formation in the pollen wall. Nat Commun.

[16] Colcombet J, Boisson-Dernier A, Ros-Palau R, Vera CE, Schroeder JI (2005). *Arabidopsis SOMATIC EMBRYOGENESIS RECEPTOR KINASES1* and *2* are essential for tapetum development and microspore maturation. Plant Cell.

[17] Jiang J, Zhang Z, Cao J (2013). Pollen wall development: the associated enzymes and metabolic pathways. Plant Biol.

[18] Yang ZF, Liu L, Sun LP, Yu P, Zhang PP, Abbas A, Xiang XJ, Wu WX, Zhang YG, Cao LY, Cheng SH (2019). *OsMS1* functions as a transcriptional activator to regulate programmed tapetum development and pollen exine formation in rice. Plant Mol Biol.

[19] Zini LM, Galati GB, Solís SM, Ferrucci MS (2012). Anther structure and pollen development in Melicoccus lepidopetalus (Sapindaceae): an evolutionary approach to dioecy in the family. Flora-Morphol Distribut Function Ecol Plants.

[20] Zhang W, Sun YJ, Timofejeva LM, Chen CB, Grossniklaus U, Ma H (2006). Regulation of *Arabidopsis* tapetum development and function by *DYSFUNCTIONAL TAPETUM1* (*DYT1*) encoding a putative bHLH transcription factor. Development..

[21] Ito T, Nagata N, Yoshiba Y, Takagi MO, Ma H, Shinozaki K (2007). Arabidopsis MALE STERILITY1 encodes a PHD-type transcription factor and regulates pollen and tapetum development. Plant Cell.

[22] Yang CY, Barrena GV, Conner K, Wilson ZA (2007). *MALE STERILITY1* is required for tapetal development and pollen wall biosynthesis. Plant Cell.

[23] Feng BM, Lu DH, Peng YB, Sun YJ, Ning G, Ma H (2012). Regulation of the *Arabidopsis* anther transcriptome by *DYT1* for pollen development. Plant J.

[24] Zhu J, Lou Y, Xu XF, Yang ZN (2011). A genetic pathway for tapetum development and function in *Arabidopsis*. J Integr Plant Biol.

[25] Xu J, Yang CY, Yuan Z, Zhang DS, Gondwe MY, Ding ZW, Liang WQ, Zhang DB, Wilson ZA (2010). The *ABORTED MICROSPORES* regulatory network is required for postmeiotic male reproductive development in *Arabidopsis thaliana*. Plant Cell.

[26] Wei MM, Song MZ, Fan SL, Yu SX (2013). Transcriptomic analysis of differentially expressed genes during anther development in genetic male sterile and wild type cotton by digital gene-expression profiling. BMC Genomics.

[27] Wu YL, Min Y, Wu ZC, Yang L, Zhu LF, Yang XY, Yuan DJ, Guo XP, Zhang XL (2015). Defective pollen wall contributes to male sterility in the male sterile line 1355A of cotton. Sci Rep.

[28] Liu F, Ma LH, Wang YW, Li YJ, Zhang XY, Xue F, Nie XH, Zhu QH, Sun J (2019). *GhFAD2-3* is required for anther development in *Gossypium hirsutum*. BMC Plant Biol.

[29] Gu JN, Zhu J, Yu Y, Teng XD, Lou Y, Xu XF, Liu JL, Yang ZN (2014). *DYT1* directly regulates the expression of *TDF1* for tapetum development and pollen wall formation in *Arabidopsis*. Plant J.

[30] Yang Y, Bao SY, Zhou XH, Liu J, Zhuang Y (2018). The key genes and pathways related to male sterility of eggplant revealed by comparative transcriptome analysis. BMC Plant Biol.

[31] Chen L, Liu YG (2014). Male sterility and fertility restoration in crops. Annu Rev Plant Biol.

[32] Bohra A, Jha UC, Adhimoolam P, Bisht D, Singh NP (2016). Cytoplasmic male sterility (CMS) in hybrid breeding in field crops. Plant Cell Rep.

[33] Wan LL, Zha WJ, Cheng XY, Liu C, Lv L, Liu CX, Wang ZQ, Du B, Chen RZ, Zhu LL, He GC (2011). A rice β-1,3-glucanase gene *Osg1* is required for callose degradation in pollen development. Planta..

[34] Ariizumi T, Toriyama K (2012). Genetic regulation of sporopollenin synthesis and pollen exine development. Annu Rev Plant Biol.

[35] Balk J, Leaver CJ (2001). The *PET1-CMS* mitochondrial mutation in sunflower is associated with premature programmed cell death and cytochrome c release. Plant Cell.

[36] Sorensen AM, Kröber S, Unte US, Huijser P, Dekker K, Saedler H (2003). The *Arabidopsis ABORTED MICROSPORES* (*AMS*) gene encodes a MYC class transcription factor. Plant J.

[37] Chen H, Li H, Gao JF, Jiang H, Wang C, Guan YF, Yang ZN (2008). *Defective in Tapetal development and function 1* is essential for anther development and tapetal function for microspore maturation in *Arabidopsis*. Plant J.

[38] Cai CF, Zhu J, Lou Y, Guo ZL, Xiong SX, Wang K, Yang ZN (2015). The functional analysis of *OsTDF1* reveals a conserved genetic pathway for tapetal development between rice and *Arabidopsis*. Sci Bull.

[39] Hu LF, Liang WQ, Yin CS, Cui X, Zong J, Wang X, Hu JP, Zhang DB (2011). Rice *MADS3* regulates ROS homeostasis during late anther development. Plant Cell.

[40] Xie HT, Wan ZY, Li S, Zhang Y (2014). Spatiotemporal production of reactive oxygen species by NADPH oxidase is critical for tapetal programmed cell death and pollen development in *Arabidopsis*. Plant Cell.

[41] Gechev TS, Van Breusegem F, Stone JM, Denev I, Laloi C (2006). Reactive oxygen species as signals that modulate plant stress responses and programmed cell death. Bioessays..

[42] Alvarado V, Thomas T. A Tapetal Specific NAC transcription factor involved in pollen development. Madison: 16th International Conference on *Arabidopsis* Research. Madison: University of Wisconsin; 2005.

[43] Distelfeld A, Pearce SP, Avni R, Scherer B, Uauy C, Piston F, Slade A, Zhao RR, Dubcovsky J (2012). Divergent functions of orthologous NAC transcription factors in wheat and rice. Plant Mol Biol.

[44] Zhang TZ, Hu Y, Jiang WK, Fang L, Guan XY, Chen JD, Zhang JB, Saski CA, Scheffler BE, Stelly DM (2015). Sequencing of allotetraploid cotton (*Gossypium hirsutum* L. acc. TM-1) provides a resource for fiber improvement. Nat Biotechnol.

[45] Trapnell C, Williams BA, Pertea G, Mortazavi A, Kwan G, Baren MJ, Salzberg SL, Wold BJ, Pachter L (2010). Transcript assembly and quantification by RNA-Seq reveals unannotated transcripts and isoform switching during cell differentiation. Nat Biotechnol.

[46] Young MD, Wakefield MJ, Smyth GK, Oshlack A (2010). Gene ontology analysis for RNA-seq: accounting for selection bias. Genome Biol.

[47] Langfelder P, Horvath S (2008). WGCNA: an R package for weighted correlation network analysis. BMC Bioinformatics.

[48] Yang L, Wu YL, Zhang M, Zhang JF, Stewart JM, Xing CZ, Wu JY, Jin SX (2018). Transcriptome, cytological and biochemical analysis of cytoplasmic male sterility and maintainer line in CMS-D8 cotton. Plant Mol Biol.

[49] Cheng XQ, Zhu XF, Tian WG, Cheng WH, Hakim SJ, Jin SX, Zhu HG (2017). Genome-wide identification and expression analysis of polyamine oxidase genes in upland cotton (*Gossypium hirsutum* L.). Plant Cell Tiss Org.

