# Supplementary figures and images for: Characterization and transcriptome analysis of a dominant genic male sterile cotton mutant
Source: BMC Plant Biol. 2020 Jul 3;20:312. doi: 10.1186/s12870-020-02522-0 (PMC7333317; doi:10.1186/s12870-020-02522-0)

**
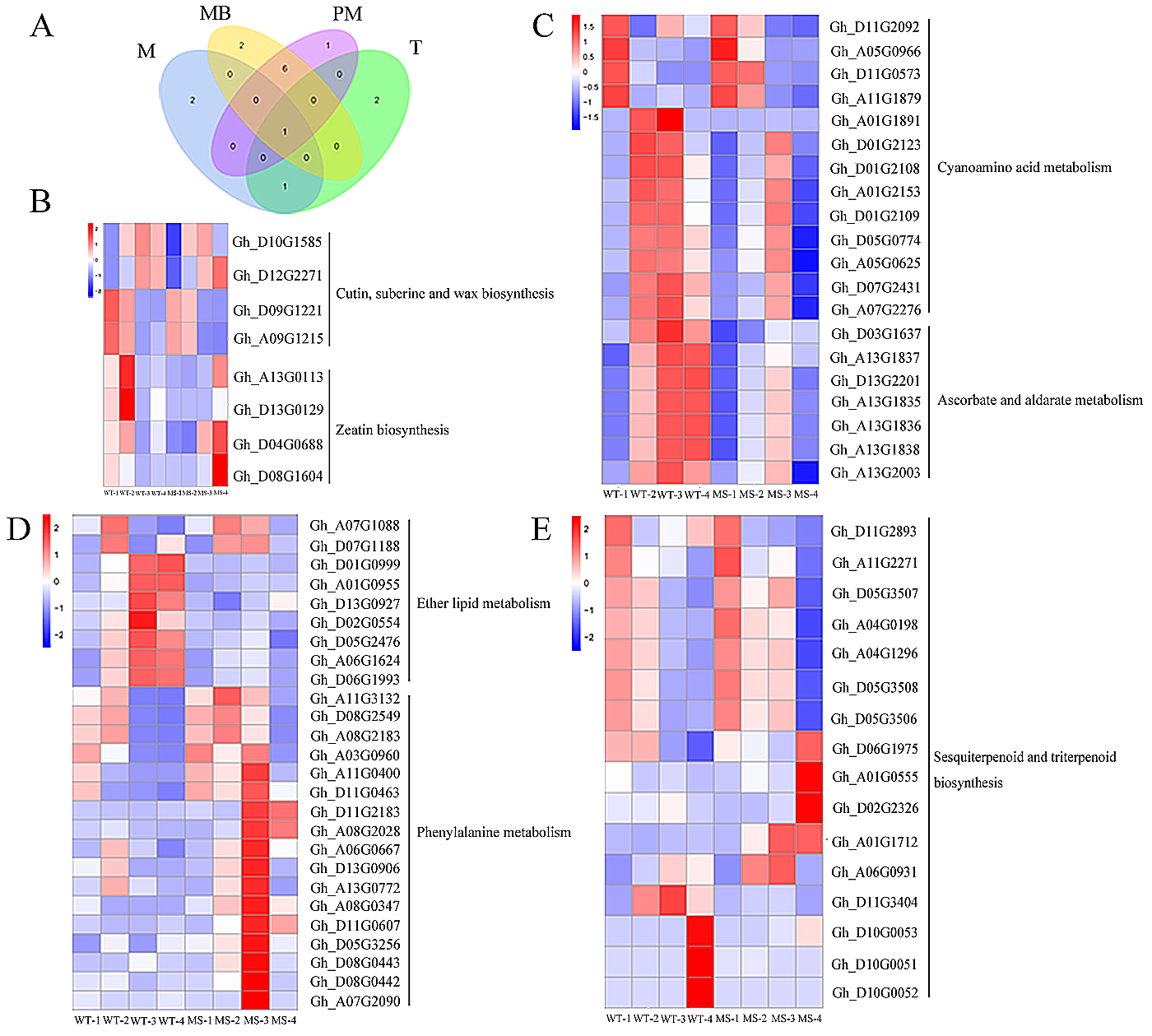
**

Fig. S1

Supplement: Supplementary file 1 — Additional file 1: Figure S1. Heatmap comparison DEGs associated with anther developmental stages. a. Venn diagram showed the different KEGGs in four stages. b, c, d and e showed the DEGs associated to the functional categories at meiosis stage, tetrad stage, mononuclear and binuclear pollen stage and pollen maturation stage, respectively. Red represents high expression, and blue represents low expression. Each row represents a DEG. [file 12870_2020_2522_MOESM1_ESM.doc]

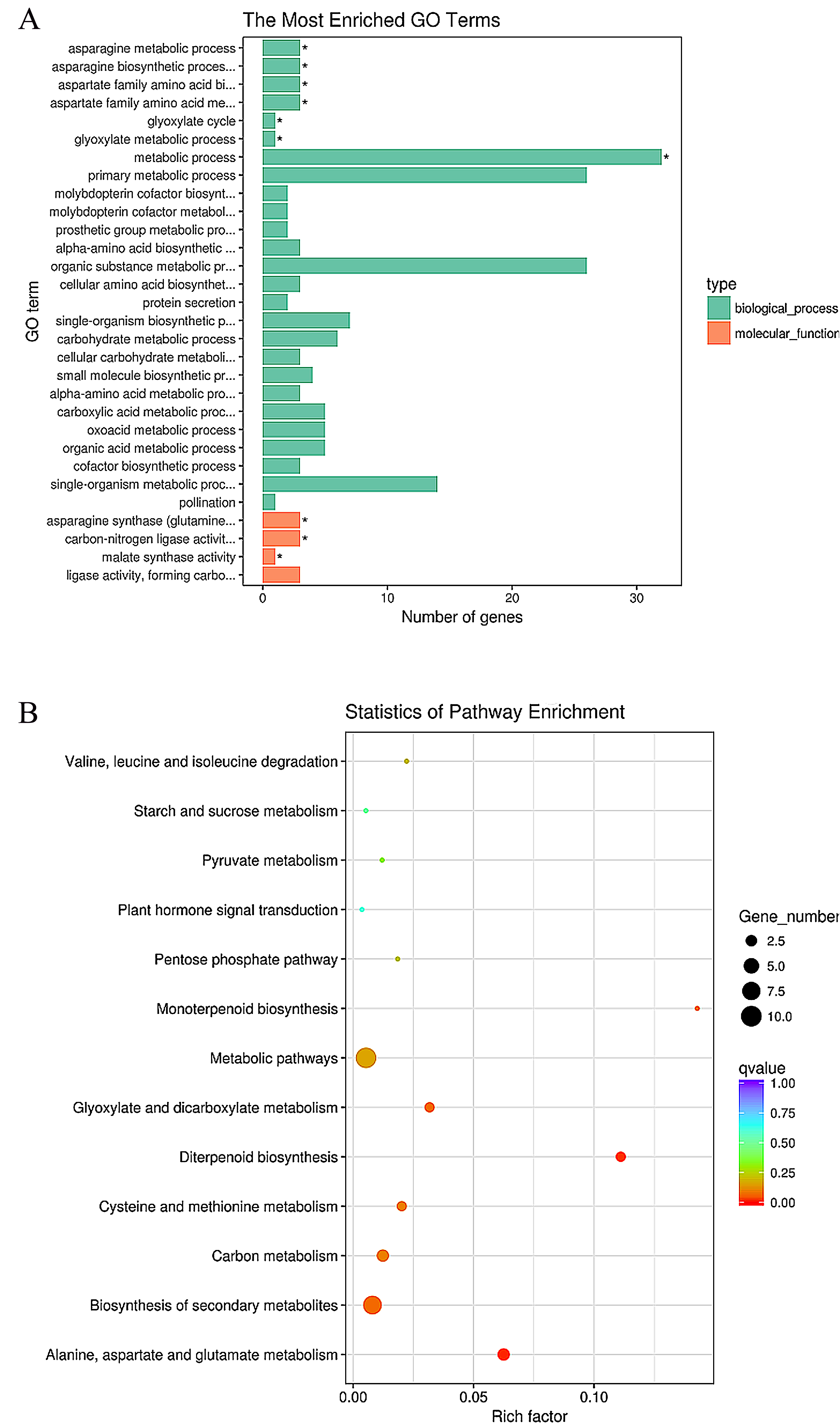


Fig. S2

Supplement: Supplementary file 2 — Additional file 2: Figure S2. GO analysis and KEGG pathways of the greenyellow module genes. a. Analysis of GO enrichment of the greenyellow module genes. * represented significant enrichment (Corrected PValue ≤ 0.05). b. KEGG categories of DEGs at the greenyellow module. The horizontal axis was rich factor, The ertical axis was statistics of pathway enrichment. Circle size represented the number of genes. Red represents high KEGG enrichment, and blue represents low KEGG enrichment. [file 12870_2020_2522_MOESM2_ESM.doc]
