# Supplementary material for: Characterization and transcriptome analysis of a dominant genic male sterile cotton mutant
Source: BMC Plant Biol. 2020 Jul 3;20:312. doi: 10.1186/s12870-020-02522-0 (PMC7333317; doi:10.1186/s12870-020-02522-0)
Supplement: Supplementary file 3 — Additional file 3: Table S1. Transcriptome sequencing data quality and genome mapping. [file 12870_2020_2522_MOESM3_ESM.doc]

Table S1

|  | Sample name | Raw reads | Clean reads | Q30  (%) | GC content  (%) | Total mapped | (%) of Total mapped | (%) of Multiple mapped | (%) of Uniquely mapped |
| --- | --- | --- | --- | --- | --- | --- | --- | --- | --- |
| meiosis | WT_1a | 50,962,200 | 50,372,704 | 93.27 | 43.13 | 48,371,638 | 96.03 | 6.16 | 89.86 |
|  | WT_1b | 51,371,980 | 50,828,322 | 92.66 | 43.15 | 48,720,076 | 95.85 | 5.99 | 89.87 |
|  | WT_1c | 48,880,124 | 48,132,986 | 93.11 | 43.1 | 46,170,252 | 95.92 | 6.39 | 89.53 |
|  | MS_1a | 49,024,804 | 48,368,976 | 93.12 | 43.25 | 46,518,423 | 96.17 | 6.23 | 89.95 |
|  | MS_1b | 51,187,708 | 50,453,498 | 93.11 | 43.31 | 48,383,217 | 95.9 | 6.5 | 89.39 |
|  | MS_1c | 48,366,102 | 47,740,720 | 93.38 | 43.36 | 45,931,142 | 96.21 | 6.67 | 89.54 |
| tetrad | WT_2a | 53,667,322 | 52,891,740 | 93.18 | 43.17 | 50,759,162 | 95.97 | 6.1 | 89.86 |
|  | WT_2b | 53,917,728 | 53,364,588 | 92.45 | 43.12 | 51,136,165 | 95.82 | 5.75 | 90.08 |
|  | WT_2c | 53,038,118 | 52,407,618 | 92.59 | 43.19 | 50,401,080 | 96.17 | 5.98 | 90.19 |
|  | MS_2a | 52,049,582 | 51,442,500 | 93.13 | 43.29 | 49,485,012 | 96.19 | 5.62 | 90.57 |
|  | MS_2b | 45,779,852 | 45,240,092 | 92.71 | 43.29 | 43,508,627 | 96.17 | 5.66 | 90.51 |
|  | MS_2c | 42,250,212 | 41,779,672 | 92.5 | 43.31 | 40,135,283 | 96.06 | 5.69 | 90.38 |
| mononuclear and binuclear pollen | WT_3a | 50332744 | 49763230 | 92.86 | 43.41 | 47775123 | 96 | 7.5 | 88.51 |
|  | WT_3b | 51875938 | 50983728 | 92.91 | 43.38 | 48828038 | 95.77 | 7.49 | 88.28 |
|  | WT_3c | 57254106 | 56262292 | 92.45 | 43.31 | 53916450 | 95.83 | 7.66 | 88.17 |
|  | MS_3a | 48798612 | 47581202 | 93.05 | 43.18 | 45553657 | 95.74 | 6.1 | 89.64 |
|  | MS_3b | 47393106 | 46713730 | 93.25 | 43.43 | 45000959 | 96.33 | 6.04 | 90.3 |
|  | MS_3c | 58407052 | 57574140 | 93 | 43.31 | 55426096 | 96.27 | 6.14 | 90.13 |
| pollen mature | WT_4a | 51,749,496 | 51,198,006 | 93.16 | 43.35 | 49,279,278 | 96.25 | 7.64 | 88.61 |
|  | WT_4b | 55,158,922 | 54,338,106 | 92.93 | 43.17 | 52,227,919 | 96.12 | 7.46 | 88.65 |
|  | WT_4c | 51,505,330 | 50,792,074 | 93.29 | 43.36 | 48,891,115 | 96.26 | 7.87 | 88.38 |
|  | MS_4a | 47,707,720 | 47,117,468 | 93.32 | 42.9 | 45,329,703 | 96.21 | 5.76 | 90.44 |
|  | MS_4b | 45,236,178 | 44,522,856 | 92.32 | 42.86 | 42,674,121 | 95.85 | 6.02 | 89.83 |
|  | MS_4c | 47,099,600 | 46,426,324 | 93.2 | 43.14 | 44,657,892 | 96.19 | 6.32 | 89.87 |
|  | Total | 1,161,509,206 | 1,145,504,498 | 92.94 | 43.22 | 1,100,189,313 | 96.04 | 6.39 | 89.66 |
